# Obstetric anaesthesia: Widening horizons?

**DOI:** 10.4103/0019-5049.71023

**Published:** 2010

**Authors:** Sunanda Gupta

**Affiliations:** President, Association of Obstetric Anaesthesiologists, RNT Medical College, Udaipur, India E-mail: sunandagupta@hotmail.com

Maternal mortality has emerged as one of the most challenging healthcare issues in the last decade, which has become an important criterion in assessing a country’s position on the development ladder. Global initiatives and concentrated efforts in this direction have started bearing fruit, as reduction in maternal mortality has been included as the fifth of the eight goals for development in the Millenium Declaration (Millenium Development Goal MDG5). The need for accurate monitoring of maternal mortality has long been recognised, both to advocate for resources and policy attention, and to track progress.[1] The current global maternal mortality is approximately 400 per 100,000 deliveries with a range of 7-740 deaths per 100,000, demonstrating the inequality between rich and poor countries. In a recent study, published in Lancet in May 2010, Hogan and colleague,[2] have reported a substantial decline in maternal mortality in 181 countries from 1980 to 2008, especially in Egypt, Romania, Bangladesh, India and China. In Asia, India and Indonesia have achieved substantially different rates of decline. The study reveals that presently India has reduced its MMR from 677 in 1980 (Indonesia: 423) to an MMR of 254 (154-395) in 2008 showing a yearly rate of decline of 4.00%, which is way ahead of Indonesia, which has shown a yearly decline of only 0.6% (423 in 1980) resulting in similar MMRs at present. However, the authors concede that, for countries which have data from several sources, such as India, there can be substantial non-sampling error across data sources.

Obstetric anaesthesia practice has had an important influence on maternal mortality. Anaesthesiologists, with their wide experience in advanced life support and resuscitation, organ support, pain management and as physicians with a thorough knowledge of maternal physiology, play a key role in the multidisciplinary team that is required to care for the critically ill obstetric patient. Pregnant and postpartum women form upto 7% of admissions in Indian Intensive care units (ICU) especially in public and private hospitals[3,4] extending up to 10%[5] in some of the low-resource countries, as against 0.1-0.9% in the developed countries.[6]

Critical care in obstetrics still remains a neglected area, with a paucity of data originating from existing obstetric critical care units in the country. Leading obstetric reasons for patients requiring critical care are pregnancy-induced hypertensive disorders and massive obstetric haemorrhage with most women being admitted in the postpartum period. Rational resource utilization and the prohibitive role of economics, demand a quantification of disease severity prediction of patient outcome in the critical care units. The catastrophic nature of obstetric emergencies in an apparently healthy parturient and the self-limiting nature of some obstetric pathologies, like pre-eclampsia and haemorrhage, following adequate and timely management, leads to overestimation or underestimation of the outcome prediction scores. Thus, an urgent modification of the traditional scoring tools and outcome predictors is required for the obstetric patients, especially keeping in mind the specific maternal problems in developing countries like India[7,8] which needs to be validated in multicentric trials. Assessment and optimization of the patient during pre-anaesthetic evaluation, robust referral systems, allowing staff to refer high-risk parturients to senior on-call anaesthetists are some of the pre-emptive strategies which could avoid requirement of a High Dependency Unit (HDU) / ICU admission.

Reducing the incidence of criticality in obstetrics is challenging to the obstetric care providers. It has clinically been established that catastrophic deterioration of patients in hospital wards is frequently preceded by documented deterioration of physiological parameters.[9,10] The early recognition of the acutely deteriorating patient using the track and trigger early-warning systems (EWS) incorporate a variety of physiological variables in scoring, and trigger algorithms.

The modified early warning scores (MEWS) system is a modified version of the EWS system,[11] which adds two parameters: measurement of urine output and deviations of blood pressure, along with heart rate, oxygen saturation, ventilatory frequency, temperature and level of consciousness. If the total score exceeds a threshold score, it triggers an immediate call for senior consultant, with simple measures to be implemented in the wards with additional monitoring of the situation or transfer to a HDU/ICU. Positive implications of usefulness of this system has been reported[8,12,13] along with criticisms about validity, reliability and sensitivity of these scores with major concerns over their accuracy.[14,15] In future, we need to work towards modifying these scores for the obstetric patient, with a new terminology, modified obstetric early warning scores (MOEWS).

The Confidential Enquiries into Maternal Deaths (CEMD) has made a number of recommendations like early consultation (CEMD 2000-2002) with senior obstetricians, anaesthetists, physicians and intensivists, when it becomes apparent that the patient is deteriorating, use of EWS in all hospital wards, adherence to obstetric emergency protocols, early identification and targeted management of sepsis and resuscitation skills training of all obstetric staff.[5]

In a country like India, where majority of the hospitals work under financial and manpower constraints, MEWS, could represent the best attempt to identify parturients at risk, and also help in stabilizing and optimizing the parturient before shifting to the HDU or ICU. There is little effort and no additional cost required to train existing paramedical staff and clinicians in recognizing the MEWS. The incremental expenditure accrued by training newly recruited additional staff would, in the long run, save on ICU costs.

Obstetric audits have had a substantial influence on shaping obstetric anaesthetic practice. The integral role of obstetric anaesthesia in peripartum and multidisciplinary obstetric care have emphasized the impact of such audits on provision of quality services by anaesthetists, intensivists and pain medicine specialist. The performance of an audit requires the establishment of a framework for the collection of national obstetric data. Developing countries like India, face challenges in collection of such data due to financial, computer and manpower constraints. Extrapolation of data from the developed countries face major barriers, as outcome, quality indicators and problems specific to the patient population differ in the developing countries. Moreover, in the developed world, the focus has shifted from maternal mortality to serious maternal morbidity (‘near misses’) which is up to 50 times more common than mortality in developed countries. Maternal mortality has fallen to such low rates, that analysis of cases of severe maternal morbidity is necessary to provide sufficient numbers to give a clinically relevant assessment of standard maternal care.[16]

The first triennial confidential enquiries started in England and Wales in 1952, has had a profound influence on the practice of obstetric anaesthesia. The recommendations from this UK triennial report which pinpointed ‘failed airway management’ as a predominant cause of anaesthetic-related maternal mortality, led to the increased use of regional anaesthesia for Caesarean Sections (CS). This significant change-over in the technique of anaesthesia for CS has brought about an ‘auditable best practice’ standard in UK that states that 95% of elective CS and 85% of emergency CS should be performed under regional anaesthesia. Some of the other audits that have had a profound impact on anaesthesia services are a ‘decision to delivery’ interval of 30 min for optimum fetal and maternal outcome, maternal morbidity and critical incident audits, deficiencies in obstetric haemorrhage management, postpartum neurological complications, neonatal outcome, etc.[16,17]

Doctors in UK are expected to perform an audit in the first 2 years following graduation.[18] In our country, a strong centralized government leadership system should formulate quality-improvement strategies, form guidelines to set up a strong database for audit, provide training to healthcare professionals in the audit process and make it mandatory for anaesthesiologists to conduct audits in tertiary care hospitals and medical colleges, to establish a framework for the collection of national obstetric anaesthetic data. Till date, we have been relying on data from the developed nations. How much they are applicable in the Indian scenario is a matter of debate.

Thus, apart from playing a key role in management of high-risk pregnancies, anaesthesiologists must be a member of the multidisciplinary team that is required to care for the critically ill parturient, educate and train the obstetric care providers in MEWS, resuscitation training, running ‘skill drills’ for emergency simulations, risk management and ensure audit of maternal morbidity and mortality.[19] All this highlights the urgent need for starting accredited courses for Anaesthesiologists, in Obstetric anaesthesia ranging from short certificate courses to full fellowships. On the other hand we have to contend with short-term anaesthesia training program of general doctors for emergency obstetric care to solve the shortage of specialist manpower in rural areas. The Indian Society of Anaesthesiologists, have voiced their concerns over this issue in the past few years, through pro-con debates, panel discussions and lectures in conferences and editorials in the Indian journals.[20,21] ‘Task shifting’ for provision of anaesthesia has been recently implemented in rural India. The World Health Organization defines task shifting as “ *the process whereby specific tasks are moved, where appropriate, to health workers with shorter qualifications and shorter trainings*.” However, both the initiation and implementation of task shifting have met with varied success across the South Asian countries.[22] Given the risks involved while giving anaesthesia, it is important to incorporate quality assurance and safety measures into any task-shifting program. In Srilanka, the medical officers are under direct or indirect supervision of qualified anaesthetists, while in Bangladesh and India, there is no such provision, hardly any data having been collected and analysed to compute complication rates and monitor quality in the rural areas, where these doctors practice.[23,24]

The changeover from general anaesthesia to regional anaesthesia has improved the safety profile of obstetric anaesthesia in the world. However, the latest CEMD (2005-2007) in South Africa reported 74 direct anaesthesia deaths of which 53 were associated with spinal (93% of which was deemed avoidable) and 18 with general anaesthesia. The contributing factors attributed to the high mortality, included inappropriate case selection, inadequate management of hypotension or high motor block, and failed intubation. This report underlines the fact that “spinal anaesthesia in inexperienced hands is also associated with significant maternal mortality.[25,26]”

Professional Obstetrical Associations can play a pivotal role in stimulating change in countries with a high mortality rate.[27] The Association of Obstetric Anaesthesia in India needs to set up standards of care and protocols especially in the context of local resource availability, highlighting areas of needed improvement, conduct continuing education programmes, which emphasize best practices based on scientific evidence and avoiding harmful practices, and promote audits for safe obstetric anaesthesia and analgesia in both rural and urban India!

‘Safe anaesthetic practice’ is one of the strategic milestones on the path towards safe obstetric services which needs to be crossed as we head towards 2015!
